# A Case of a 4-Year-Old Boy with a Mesenteric Chylous Cyst Infected with* Histoplasma capsulatum*

**DOI:** 10.1155/2016/4296059

**Published:** 2016-01-06

**Authors:** Vianney Kweyamba, Mirraim Apiyo, Biratu Olika, Olivia Kituuka

**Affiliations:** ^1^Department of Surgery, Case Hospital, Kampala, Uganda; ^2^Department of Pediatrics, Case Hospital, Kampala, Uganda; ^3^Gamma Path Laboratory, Kampala, Uganda

## Abstract

Mesenteric cysts are uncommon entities and chyle- (lymph-) containing cysts are the rarest of this group. This is a case report of a 4-year-old boy with a mesenteric chylous cyst who was later found to have* Histoplasma capsulatum* infection.

## 1. Introduction

Chylous cysts are benign proliferations of lymph vessels which result from an obstruction in the lymphatic system. They account for approximately 3% to 9.2% of all paediatric lymphangiomas, although their incidence is unknown [1, 2].

These lesions can present with symptoms such as abdominal pain, nausea, vomiting, anorexia, and changes in bowel habits; however, most commonly they are asymptomatic and are detected incidentally on physical exam or imaging. Although most mesenteric cysts are benign, these lesions occasionally cause complications, including intestinal obstruction, volvulus, or even torsion [3, 4].

## 2. Case Presentation

A 4-year-old boy presented to our hospital with a 6-month history of on and off vague abdominal pain and anorexia. Two weeks prior to admission, the pain remained vague but was associated with occasional nonprojectile vomiting and nausea; he however continued to have normal bowel habits. A day prior to coming to the hospital, he started having projectile Bilious Vomiting.

He was admitted as a patient of acute absolute intestinal obstruction, possibly due to intussusception. The preliminary blood work revealed no derangement in function; that is, he had normal complete blood count, liver function tests, and renal function tests. Abdominal ultrasound was done, where reduced bowel peristalsis was seen, with no other remarkable findings. The plain abdominal X-ray showed a possible gross dilation of colon in the hepatic flexure with few air-fluid levels.

**Figure 1:**
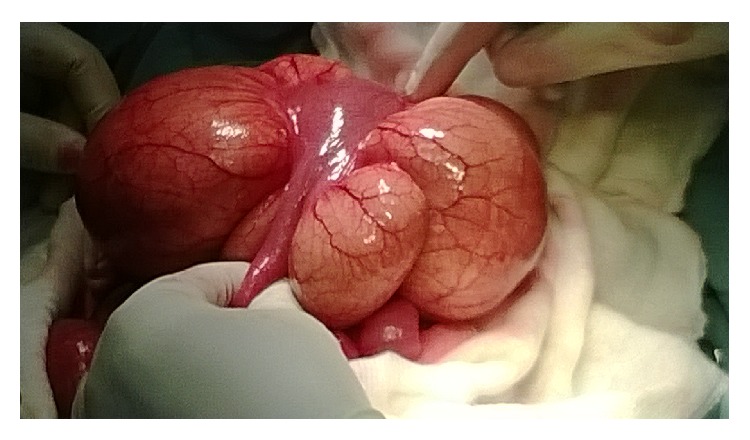
Intestine compressed but viable.

**Figure 2:**
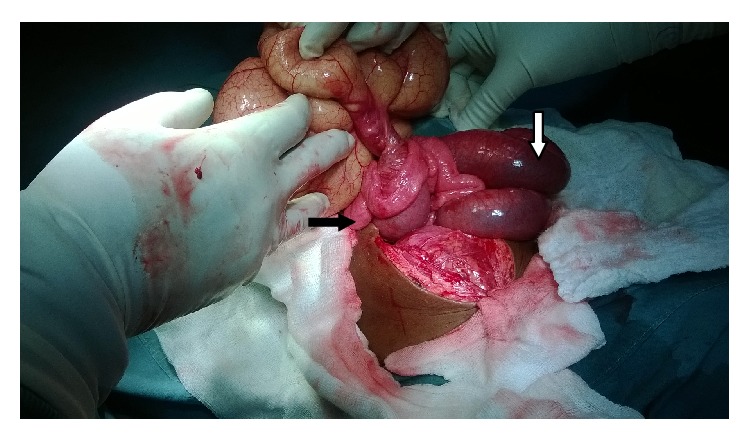
The pedicle of the cyst with proximal intestine dilation (white arrow) and distal intestine collapse (black arrow).

**Figure 3:**
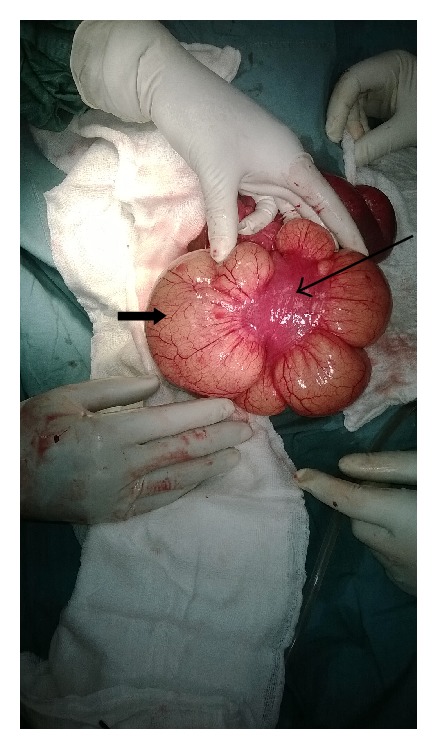
Intestine (long arrow) compressed by the cyst (short arrow).

On Exploratory Laparotomy, we found a very large (15 cm in diameter) mesenteric cyst within the small bowel mesentery located 30 cm from the ileocecal junction and obstructing a portion (10 cm) of small bowel lying above it (Figures 1, 2, and 3).

Excision of the cyst together with resection and anastomosis of the affected bowl were carried out. We drained 750 mL of chylous fluid from the excised cyst.

**Figure 4:**
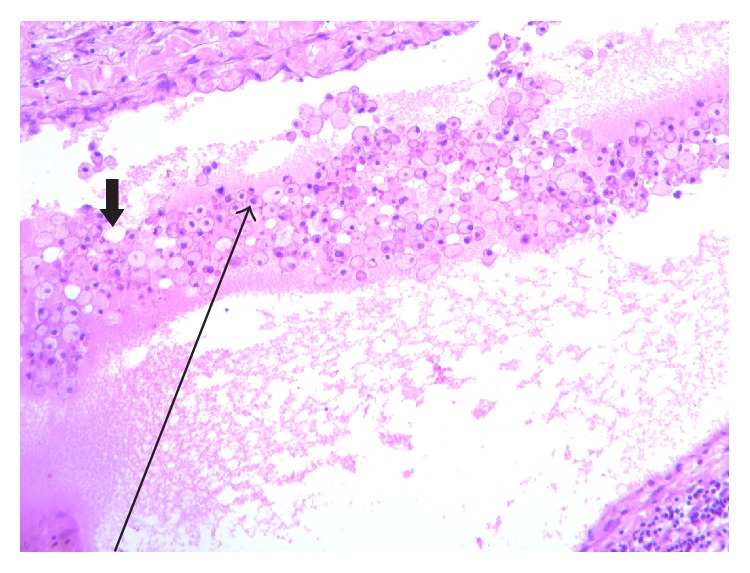
H&E stain (low magnification). Diffuse sheets of foamy macrophages (long arrow) admixed with chylous spaces (short arrow) H&E ×40.

**Figure 5:**
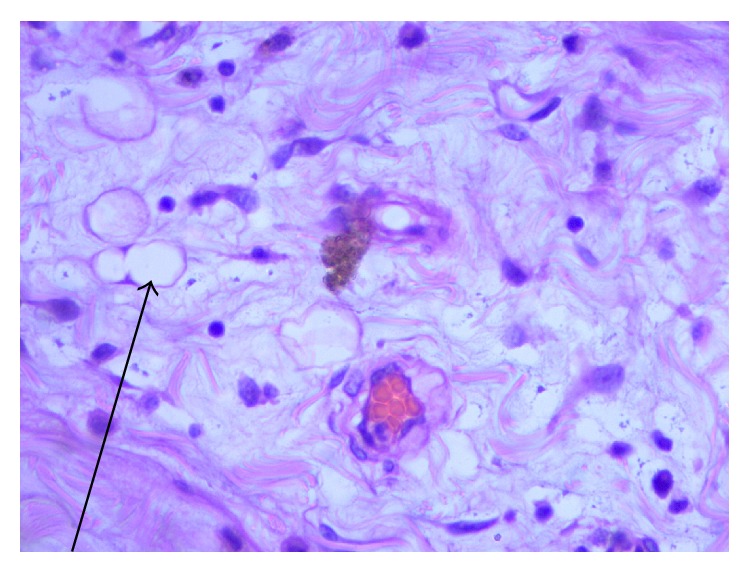
H&E stain (high magnification). Vascular spaces filled with clear chylous fluid (arrow). H&E stain ×100.

**Figure 6:**
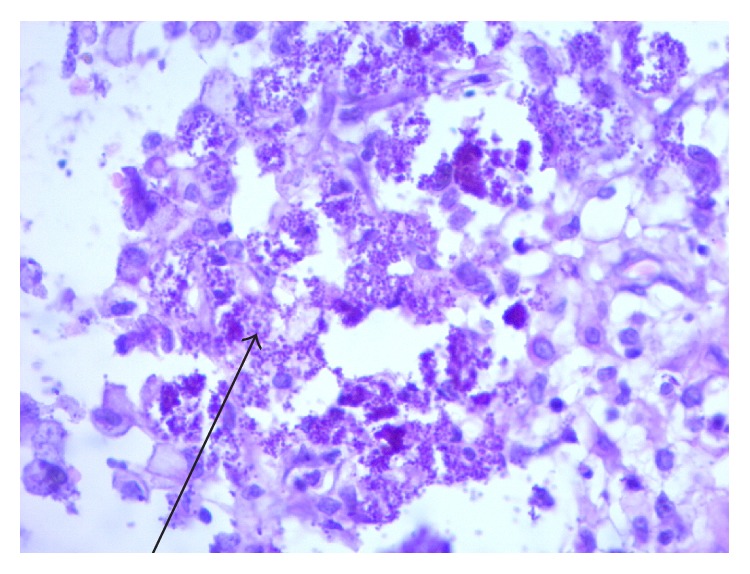
PAS fungal stain. PAS stain: depicting basophilic intracellular bodies within the macrophages (arrow) consistent with* Histoplasma capsulatum*. There is no evidence of granulomatous reaction.

Histopathological findings (Hematoxylin and Eosin stain, Ziehl-Neelsen stain, and periodic acid-Schiff stain) showed the presence of histoplasmosis spores within the lining of the cyst (Figures 4, 5, and 6).

The patient had an uneventful postoperative recovery period. He was discharged 5 days later after full recovery. Parents report that he has since gained an appetite for food. He also is currently on itraconazole treatment.

## 3. Discussion

Chylous cysts are rare variants of mesenteric lesions, making up 7.3% to 9.5% of all abdominal cysts [5] and approximately 3% to 9.2% of all paediatric lymphangiomas [1]. There are very few cases of paediatric chylolymphatic cysts reported in the literature [5].

According to Beahrs et al. [6], based on etiology, mesenteric cysts can be classified into four groups, that is, embryonic/developmental, traumatic/acquired, neoplastic, and infective/degenerative. Classification based on pathology has also been proposed [7], where type 1 (pedicled) and type 2 (sessile) cysts are limited to the mesentery and can be excised completely with or without resection of the involved gut and types 3 and 4 (multicentric) cysts extend into the retroperitoneum and require complex operations often with sclerotherapy as well.

Furthermore, based on the contents of the cyst, the mesenteric cysts can also be classified into serous, chylous, hemorrhagic, and chylolymphatic cysts. The chylolymphatic cysts, as indicated by the name, contain both chyle and lymph. Therefore, in this patient, the cyst can be classified as an* infective, type 2, chylous cyst*.

These cysts can present with symptoms such as abdominal pain, nausea, vomiting, anorexia, and a change in bowel habits; however, most commonly they are asymptomatic and are detected incidentally on physical exam or imaging. Even though most mesenteric cysts are benign, these lesions do occasionally cause complications, including intestinal obstruction, volvulus, or even torsion [3, 4]. In the case presented, the patient came in with a presentation of intestinal obstruction.

The preoperative diagnosis may be achieved with the common imaging examinations of the abdomen (ultrasonography, abdominal X-ray, and computed tomography). Ultrasonography usually demonstrates a cystic tumour whose content may form a fluid-fluid level in case of a chylolymphatic cyst [8]. A plain abdominal X-ray may show a gasless, homogenous mass defect displacing the bowel loops around it. In a child with an obstructed intestine, multiple air-fluid levels will be seen on an erect abdominal X-ray [5]. Computed tomography scans show a cystic mass with a thick wall and a fluid content with a low CT number [8]. Abdominal imaging is particularly useful to demonstrate the relation of the tumour to the major abdominal vessels to adequately plan the surgical approach. Laboratory tests may be done in addition to imaging to rule out other possible causes of cystic lesions like mesenteric TB abscesses. In this patient only the abdominal ultrasound and a plain abdominal X-ray were carried out, given the urgency to intervene.

Different surgical approaches are used, that is, marsupialization, sclerotherapy, drainage, enucleation, percutaneous aspiration, and excision of the cyst with or without resection of the involved gut [9–12]. But due to high recurrence rates associated with marsupialization and drainage, complete excision of the cyst should be attempted whenever possible [10]. In adults, the cyst can often be enucleated or “shelled out” from between the leaves of the mesentery; in children, however, bowel resection is frequently required [10, 13, 14]. Bowel resection and anastomosis were done in this patient so as to completely excise the whole cyst.

Histoplasmosis is caused by inhaling the microconidia (spores) formed by the environmental mold. Healthy hosts, unless they inhale many conidia, remain asymptomatic or have only mild pulmonary symptoms when exposed to* H. capsulatum*.* H. capsulatum* is one of several thermally dimorphic fungi; at 35–37°C in the lungs, it transforms from a mold state into a yeast state [15].

Before immunity develops, in almost all cases, the organism is disseminated by the macrophages moving to local mediastinal and hilar lymph nodes and, more widely, to the liver, spleen, lymph nodes, and other organs. This dissemination is asymptomatic in most individuals but can lead to progressive disseminated infection in people that are immunosuppressed [16].

All gastrointestinal (GI) forms of histoplasmosis are likely manifestations of disseminated disease. Acquisition by the GI route through water or food ingestion has been suggested, but this has never been proven and remains a very unlikely* possibility* [15].* H. capsulatum* can be found throughout the GI tract from the mouth to the anus [17]. It is likely that lymph nodes throughout the GI tract, especially the numerous lymph nodes in the ileocecal region, are seeded during hematogenous dissemination.

The most common presenting symptoms of GI histoplasmosis are abdominal pain and diarrhea [18, 19]. Diarrhea is often intermittent and similar to that seen in many other diseases. Many patients have only mild symptoms of abdominal pain and occasional diarrhea but have prominent systemic manifestations of disseminated histoplasmosis, including fevers, night sweats, weight loss, and fatigue. In our case there were no signs indicating disseminated histoplasmosis; the patient was not immunosuppressed.

Patients who have mild-to-moderate disease can be treated with oral itraconazole as initial therapy. Itraconazole is best administered as an oral solution at a dosage of 200 mg three times daily for 3 days as a loading dose and then twice daily for 12 months. Itraconazole levels should be monitored after the second week of therapy to be certain that serum levels are at least 2 *µ*g/mL. The oral solution is given on an empty stomach. If the patient cannot tolerate the solution, capsules can be substituted. Itraconazole capsules must be given with food and require acid for absorption, so acid-inhibiting drugs cannot be prescribed for these patients [20].

Most patients with disseminated histoplasmosis respond well to antifungal therapy. This patient is currently on treatment.

## 4. Conclusion

We cannot confirm whether the chylous cyst was due to the histoplasmosis infection or whether the histoplasmosis secondarily colonized an already existent cyst. But the presentation of these two conditions in the same patient makes it a very rare presentation.
